# Early Degenerative Changes in a Spontaneous Osteoarthritis Model Assessed by Nanoindentation

**DOI:** 10.3390/bioengineering10090995

**Published:** 2023-08-23

**Authors:** Sarah Davis, Jurgita Zekonyte, Aikaterina Karali, Marta Roldo, Gordon Blunn

**Affiliations:** 1 School of Pharmacy and Biomedical Science, University of Portsmouth, Portsmouth PO1 2DT, UK; marta.roldo@port.ac.uk (M.R.); gordon.blunn@port.ac.uk (G.B.); 2 School of Mechanical and Design Engineering, University of Portsmouth, Portsmouth PO1 3DJ, UK; jurgita.zekonyte@port.ac.uk (J.Z.); katerina.karali@port.ac.uk (A.K.)

**Keywords:** nanoindentation, osteoarthritis, osteochondral interface, articular cartilage, subchondral bone, mechanical properties

## Abstract

Understanding early mechanical changes in articular cartilage (AC) and subchondral bone (SB) is crucial for improved treatment of osteoarthritis (OA). The aim of this study was to develop a method for nanoindentation of fresh, unfixed osteochondral tissue to assess the early changes in the mechanical properties of AC and SB. Nanoindentation was performed throughout the depth of AC and SB in the proximal tibia of Dunkin Hartley guinea pigs at 2 months, 3 months, and 2 years of age. The contralateral tibias were either histologically graded for OA or analyzed using immunohistochemistry. The results showed an increase in the reduced modulus (E_r_) in the deep zone of AC during early-stage OA (6.0 ± 1.75 MPa) compared to values at 2 months (4.04 ± 1.25 MPa) (*** *p* < 0.001). In severe OA (2-year) specimens, there was a significant reduction in E_r_ throughout the superficial and middle AC zones, which correlated to increased ADAMTS 4 and 5 staining, and proteoglycan loss in these regions. In the subchondral bone, a 35.0% reduction in stiffness was observed between 2-month and 3-month specimens (*** *p* < 0.001). The severe OA age group had significantly increased SB stiffness of 36.2% and 109.6% compared to 2-month and 3-month-old specimens respectively (*** *p* < 0.001). In conclusion, this study provides useful information about the changes in the mechanical properties of both AC and SB during both early- and late-stage OA and indicates that an initial reduction in stiffness of the SB and an increase in stiffness in the deep zone of AC may precede early-stage cartilage degeneration.

## 1. Introduction

Osteoarthritis (OA) is a chronic degenerative disease that affects over 500 million people worldwide [1] and is one of the leading causes of disability resulting in pain, joint stiffness, and restricted movement. The pathogenesis of OA is not yet fully understood, and the mechanisms involved in the initiation and progression of OA, which subsequently lead to articular cartilage (AC) degeneration, still need elucidating. OA is primarily characterized by joint space narrowing [2], osteophyte formation [3,4], subchondral bone sclerosis [5,6], and synovial inflammation [7] and the interaction of multiple joint tissues is becoming of paramount importance in current OA research [8,9,10,11,12,13,14]. For example, changes to the subchondral bone including increased remodeling [5,15,16], increased bone volume [17], increased subchondral plate thickness [18], and subchondral bone sclerosis [5,6], are some of the early pathological changes observed during OA initiation. In particular, increases in bone mineral density [18,19,20], relative stiffening, decreased porosity [21], and the formation of Bone Marrow Lesions (BMLs) [22,23,24,25,26] have shown to precede early cartilage degeneration [21,25] and are therefore thought to play a crucial role in OA initiation and progression. These structural alterations to SB alter strain distribution [27] and hence the mechanical properties of the overlying AC, disrupting load transfer throughout the osteochondral unit and unbalancing joint homeostasis. However, further research is needed to determine why these bone changes occur and the effect of these changes on AC. Therefore, understanding the initial changes in the mechanical properties of SB and AC during early-stage OA warrants study. This will ultimately improve knowledge regarding early-OA pathology and allow for better-targeted treatment methods.

Mechanical properties of osteochondral tissues can be determined by (confined or unconfined) compression testing, three-point bending, or indentation (on the macro, micro, or nanoscale) [28,29,30,31]. Nanoindentation has several advantages over other forms of mechanical testing, as it measures real-time load-displacement at a submicron resolution, in small volumes of sample material, where spatially dependent heterogenous or hierarchically structured tissues (such as AC) can be distinguished [32], and provides accurate measurements of stiffness, elastic modulus, and hardness [33]. Nanoindentation has previously been used for the nanomechanical characterization of articular cartilage [34,35,36,37,38] and subchondral bone [39,40,41,42,43,44], both across the osteochondral interface [45,46,47,48] and during varying stages of OA development [29,41,49,50,51]. Previous studies have shown that both the elastic modulus and hardness of AC decrease with disease progression [35,49] and that SB elastic modulus is strongly correlated to OA grade [50]. However, not only are there conflicting results regarding changes to SB stiffness at the nanoscale, which requires further investigation [29,40,48], but few studies focus on the interrelationship between the AC and SB and the related alterations to material properties during the very initial stages of OA. Furthermore, analysis of material properties using nanoindentation throughout the whole osteochondral zone has so far been limited, due to its heterogenous architecture and the large difference in composition, mineralization, and material properties of these diverse tissue types.

Since most nanoindentation methods involve dehydration [52], fixation [53], or embedding the tissue in resin during sample preparation [36,39,47,48,54], the values so far reported may not accurately reflect the actual mechanical properties of osteochondral tissues in vivo. For example, studies have shown an increase in the indentation modulus of up to 28% in bone tissues after dehydration [55,56]. These effects are even more enhanced in other biological tissues such as dentin [57], in which the modulus has been reported to increase 100-fold following dehydration [58]. Further increase in mechanical properties of up to 66% has also been observed after embedding in resins such as polymethylmethacrylate (PMMA) [55]. To minimize the effects of tissue degradation, biological specimens are also often preserved using formalin fixation; however, this has also been shown to have a negative effect on the biomechanical properties of biological tissues by increasing the stiffness of bone [59], aortic tissue [60], tendon [61], and cartilage by increasing collagen-cross links [53,62,63]. Finally, the hydration state also has an important effect on nanoindentation results. Hard tissues such as bone, in dry conditions, can show up to an order of magnitude of difference in the elastic modulus [64], while soft or viscoelastic tissue such as cartilage or hydrogels can present an increase in stiffness of three orders of magnitude when tested in either air or ethanol [65]. Furthermore, when tested in water, bone has been shown to be more structurally compliant compared to the use of physiological saline solution [66]. Optimal and natural conditions for nanoindentation, allowing comparable quantitative results across studies of osteochondral tissues are limited. Improving nanoindentation techniques is particularly important when attempting to detect subtle changes, particularly in soft tissues, during the early stages of OA. Therefore, developing a method for specimens that are not embedded, dehydrated, or fixed and are maintained in physiological conditions using saline solution to detect the mechanical properties of osteochondral tissues representative of those in an in vivo environment would be of great benefit.

Severely degraded human OA specimens can be obtained from end-stage surgical procedures such as total joint replacement; however, obtaining osteochondral tissue that reflects early-stage OA, before symptoms and degradation become too severe, is problematic [67]. Therefore, understanding the initial changes during early-OA progression often relies on the use of animal models. This study uses Dunkin Hartley (DH) guinea pigs; a well-established model of spontaneous OA in which characteristic histological, biochemical, and radiological changes are representative of those observed in human knee OA [68,69,70,71,72,73]. These degenerative changes include chondrocyte death, proteoglycan loss, and surface fibrillation, and these characteristic features predominantly occur on the medial tibial plateau and are similar to those early changes seen in human OA [74,75,76]. Histological changes in DH guinea pigs are first reported at approximately 3 months old and the severity of OA increases with age [68]. DH guinea pigs also show evidence of early subchondral bone changes, similar to that of humans and other animal models, including subchondral bone remodeling [67], thickening of the subchondral plate [76], and subchondral bone sclerosis [68] as well as musculoskeletal aging [69]. However, few studies focus on the interrelationship between the articular cartilage and subchondral bone and the alterations to material properties during the very initial stages of OA.

Therefore, this study aims to develop a method for liquid nanoindentation in fresh unfixed osteochondral tissue to accurately assess the initial changes in the mechanical properties of AC and SB with varying degrees of OA and to compare these changes with standard histological and immunohistochemical observations during OA progression.

## 2. Materials and Methods

### 2.1. Specimen Preparation

Intact whole stifle joints from female Dunkin Hartley (DH) guinea pigs aged 2 months, 3 months, and 2 years (*n* = 3 per group) were obtained. These were hypothesized to represent pre-OA changes, early-OA changes, and severe OA degradation, respectively [77]. The left joints for each age group (*n* = 3) were stored at −80 °C prior to nanoindentation tests (Section 2.2), which were performed in triplicate to compensate for the small sample size. The contralateral right hindlimbs (*n* = 3) were fixed in 10% *v/v* Neutral-Buffered Formalin (NBF) (pH 6.90–7.10) (HT501128; Sigma-Aldrich, Burlington, MA, USA) for 2–3 days, decalcified in 10% *w/v* ethylenediaminetetraacetic acid (EDTA) (ED-1Kg, #BCCB3404, Sigma-Aldrich, Burlington, MA, USA) (pH 7.4) for 6 weeks, with the solution changed weekly, and sectioned for either histological or immunohistochemical analysis (Section 2.4 and Section 2.5).

### 2.2. Nanoindentation

**Figure 1:**
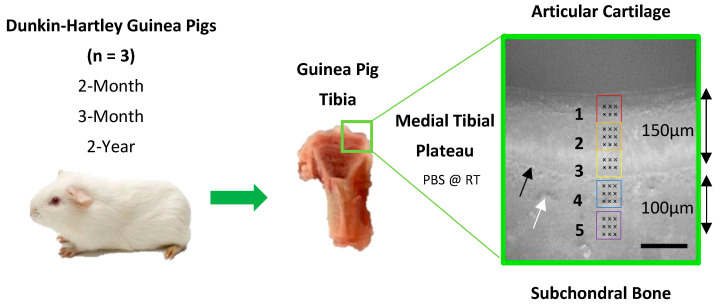
Schematic of the nanoindentation method across 5 different locations across the osteochondral interface. Locations 1 to 3 correspond to the superficial zone (SZ), middle zone (MZ), and deep zone (DZ) of articular cartilage, Location 4 includes the calcified cartilage (CC), and location 5 represents the subchondral bone (SB). Scale Bar = 100 μm. Black arrow indicates tidemark. White arrow indicates cement line. Measurements were performed in triplicate on the flat surface of both halves of the joint to compensate for the small number of animals used (*n* = 3).

Left intact stifle joints (*n* = 3) were rapidly defrosted for 1 h in PBS at room temperature to minimize degradative changes to the extracellular matrix [78] and disarticulated. The tibia was cut longitudinally with a PBS irrigated diamond annular diamond wheel (Leica SP1600 saw microtome, Leica microsystems Inc., Wetzlar, Germany). Specimens were mounted flat face up on plastic microscope slides (#333-5689-01T, Caplugs Evergreen, Rancho Dominguez, CA, USA) with Ethly-2-cyanoacrylate and reinforced with rapid-cure epoxy adhesive (ITW Devcon, Danvers, MA, USA) and indentation was performed on the flat surface of both halves (*n* = 6). Quasi-static nanoindentation (Hysitron TI Premier Nanoindenter, Bruker) using a standard diamond Berkovich tip (TI-0039, Bruker, Billerica, MA, USA) and TriboScan software (TriboScan Professional, Hysitron, Bruker, Billerica, MA, USA) was carried out across the central region of the medial tibial condyle, throughout five optically defined locations (50 × 50 µm areas) (Figure 1). These areas covered the depth of the AC (locations 1–3; corresponding to the superficial zone, middle zone, and deep zone), the calcified cartilage (CC; location 4), and the subchondral bone (SB; location 5). Nanoindentation was performed with the specimen submerged in PBS (1×, pH 7.4) at room temperature. A maximum load of 15µN was applied at a load rate of 1.5 µN/s with a 30s hold time at maximum load to allow for creep due to the viscoelasticity of articular cartilage. Multiple indentations (3 × 3 indents at locations 2, 4, and 5; or 2 × 3 indents at locations 1 and 3) were performed (Figure 1). These indentations were replicated on fresh cartilage and subchondral bone in two other areas. Raw data included force-displacement curves, in which computerized outputs of stiffness, hardness, and the reduced modulus (E_r_) were calculated using the Oliver–Pharr method [33]. The reduced modulus measures the stiffness of a combination of both the sample and the tip; however, these measurements were used for analysis rather than conversion to Young’s modulus since this assumes isotropy of the material, which is not true, particularly for the inhomogeneous structure of AC [33,79].

### 2.3. Histological Assessment of Osteoarthritis

Intact joints were cut coronally, histologically processed and paraffin wax embedded. Sections (5 μm) were mounted on Superfrost plus slides (Thermofisher, Waltham, MA, USA) and stained with either Hematoxylin and Eosin (H&E) (MH51; HT110116; Sigma-Aldrich, Burlington, MA, USA) or Toluidine Blue (0.04% in 0.2 M sodium acetate buffer, pH 4.2, 198161-5G, Sigma-Aldrich, Burlington, MA, USA) [80] and mounted with DPX (#06522; Sigma-Aldrich, Burlington, MA, USA). Images were captured using a DMi1 light microscope with an MC170 camera (Leica Microsystems Inc., Wetzlar, Germany) and scored using the semi-quantitative modified Mankin scoring system [81] by three blinded observers whose scores were averaged to produce a total histologic score.

### 2.4. Immunohistochemistry

Sections were deparaffinized in xylene and rehydrated with decreasing concentrations of ethanol. Antigen retrieval was performed at 60 °C with Tris EDTA buffer (pH 9). Hydrogen peroxide (ab64218; Abcam, Cambridge, UK) blocking solution was used followed by BSA protein block (2.5%) in PBS to block non-specific background staining. Immunohistochemical markers of OA including A Disintegrin and Metalloproteinase with Thrombospondin Motifs (ADAMTSs) were detected using rabbit anti-ADAMTS4 (1:100; ab185722; Abcam, Cambridge, UK) and rabbit anti-ADAMTS5 (1:100; ab231595; Abcam, Cambridge, UK) polyclonal antibodies. COLII was detected with rabbit Anti-Collagen II polyclonal (IgG) antibody (1:250; ab34712; Abcam, Cambridge, UK). Slides were incubated with Goat Anti-Mouse (IgG) H&L (HRP) secondary antibody (1:2000; ab205719; Abcam, Cambridge, UK) for 1 h at RT followed by DAB (3,3′Diaminobenzidine) chromogen (ab64238; Abcam, Cambridge, UK). Sections were counterstained with Mayer’s Haemaoxylin (MH51; HT110116; Sigma-Aldrich, MA, USA) mounted with DPX (#06522; Sigma-Aldrich, Burlington, MA, USA) and images were taken with a DMi1 light microscope with MC170 camera (Leica microsystems Inc., Wetzlar, Germany).

### 2.5. Statistical Analaysis

All statistical analysis was performed using GraphPad Prism 8.02 (GraphPad Software, San Diego, CA, USA). Quantitative results are expressed as mean ± SD where a *p*-value of < 0.05 was considered statistically significant. The normal distribution of all data was confirmed using Shapiro–Wilk normality tests. Comparisons between groups were analyzed using either One-way or Two-way ANOVA followed by Tukey multiple comparisons tests.

## 3. Results

### 3.1. Age-Associated Degeneration of Dunkin-Hartley Guinea Pig Joints

**Figure 2:**
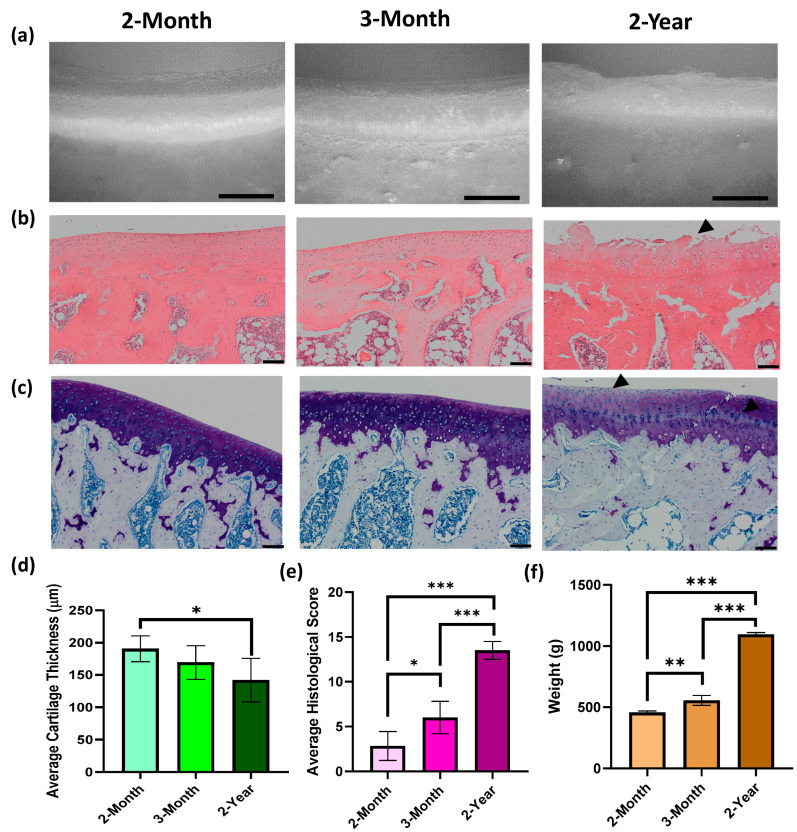
Age-associated degeneration of Dunkin Hartley Guinea Pig Joints with representative images of: (**a**) optical images, (**b**) H&E-stained sections; (**c**) and toluidine blue-stained sections with bar charts of (**d**) average AC thickness (* *p* = 0.0144); (**e**) average histological score (* *p* = 0.046; *** *p* < 0.0001) and (**f**) average body weight (** *p* = 0.0068; *** *p* < 0.001). Data are presented as mean ± SD (*n* = 3). Scale bars = 100 µm. Black arrows indicate surface fibrillation and proteoglycan depletion associated with severe OA.

The expected age-associated increase in OA degradation was observed and histologically scored from H&E and toluidine blue-stained sections using the Modified Mankin scoring system (Figure 2). In 3-month specimens, early signs of OA such as hypercellularity and clustering were observed (Figure 2b) with areas of surface fibrillation and loss of proteoglycan content (Figure 2b,c) equating to a significantly higher histological score (Figure 2e; * *p* = 0.046). In 2-year specimens, cartilage thickness had significantly decreased (Figure 2d; * *p* = 0.0144) and signs of severe OA degradation, such as deep fissures, proteoglycan loss (black arrows, Figure 2b,c), and advancement/duplication of the tidemark were evident, increasing the average total histological score (Figure 2e; *** *p* < 0.001). The body weight of DH guinea pigs also significantly increased with age (Figure 2f; ** *p* = 0.0068; *** *p* < 0.001), an important predisposing factor for the development of spontaneous OA.

### 3.2. Mechanical Alterations to Articular Cartilage during Osteoarthritis Developmen

**Figure 3:**
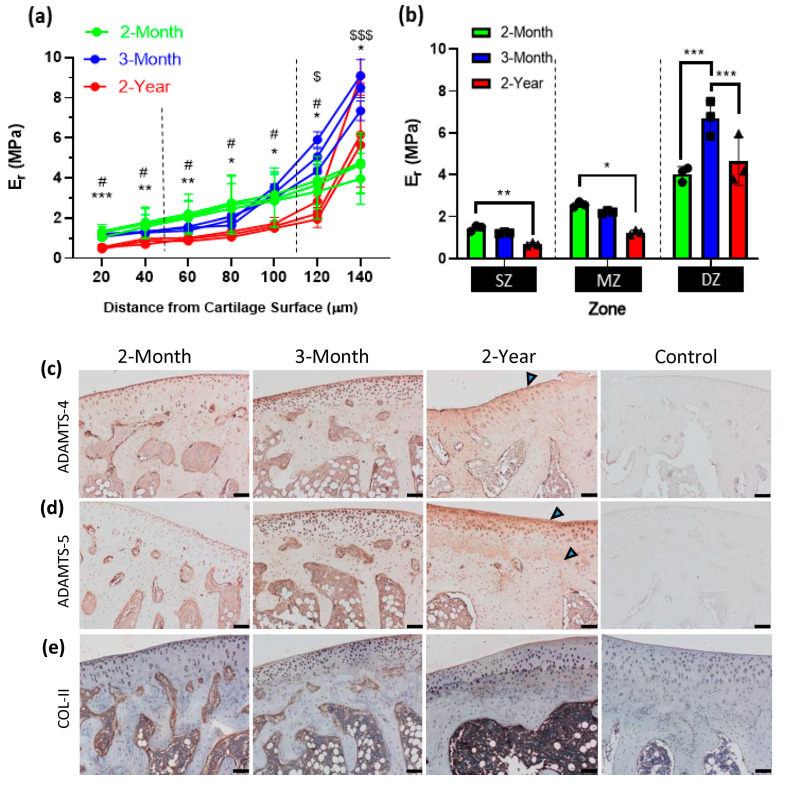
Mechanical properties of articular cartilage: (**a**) The average reduced modulus (E_r_) throughout the depth of articular cartilage for each specimen (* *p* ≤ 0.05, ** *p* ≤ 0.01, *** *p* < 0.001, 2-month compared to 2-year; # *p* < 0.05 for 3-month compared to 2-year; $ *p* = 0.039, $$$ *p* < 0.001, 2-month group compared to 3-month group). Data were plotted for each individual specimen (*n* = 3), whereas significance was calculated and displayed as averages for each age category; (**b**) bar chart displaying average E_r_ values (±SD) for all specimens and averaged for representative cartilage zone (SZ = superficial zone, MZ = middle zone, DZ = deep zone) (* *p* = 0.0116; ** *p* = 0.0032; *** *p* < 0.001)_;_ (**c**–**e**) immunohistochemistry of ADAMTS4, ADAMTS5, and COLII expression in 2-month, 3-month, and 2-year specimens. Scale bars = 100 µm. Arrows indicate areas with increased staining.

Nanoindentation throughout the AC ranged from 20 to 140 µm depth from the cartilage surface and showed a gradient increase in the average E_r_ throughout the individual specimens of all ages (Figure 3a). Severe OA in 2-year specimens had an average reduction in E_r_ values compared to 2-month (from 20–140 µm; * *p* ≤ 0.05, ** *p* ≤ 0.01, *** *p* < 0.001), and 3-month specimens (from 20–120 µm; # *p* < 0.05) (Figure 3a). A significant reduction in E_r_ was also detected between the 2-month and 3-month age groups in the deep zone (DZ, 120–140 µm) only (Figure 3a; $ *p* = 0.039, $$$ *p* < 0.001). In addition, when the data were averaged for each AC zone (Figure 3b) the average E_r_ was significantly reduced in the superficial zone (SZ) from 1.46 (±0.51) MPa in 2-month specimens to 0.68 (±0.23) MPa in 2- year specimens (** *p* = 0.0032). This trend was also observed in the middle zone (MZ) in which 2-year late-stage OA specimens had a reduced E_r_ value of 1.25 (±0.31) MPa compared to both 2-month (2.57 ± 1.08 MPa, *** *p* < 0.001) and 3-month-old specimens (2.11 ± 0.63 MPa, * *p* = 0.0289; Figure 3b). These decreases in E_r_ stiffness in the superficial and middle zones in 2-year specimens correlate with ADAMTS-4 and ADAMTS-5 staining of the extracellular matrix, in which the staining intensity is the most pronounced in the SZ and the upper MZ (Figure 3c,d; arrows). In the deep zone of AC (indentation location 3), early-stage OA 3-month specimens, had a significantly increased average E_r_ of 6.0 (±1.75) MPa compared to 2-month specimens (4.04 ± 1.25 MPa, *** *p* < 0.001), which significantly decreased during late-stage OA to 4.65 (±2.88) MPa (** *p* = 0.003) (Figure 3b). Collagen content remained unchanged throughout OA progression (Figure 3e).

### 3.3. Mechanical Alterations to Mineralized Regions of Calcified Cartilage and Subchondral Bone

**Figure 4:**
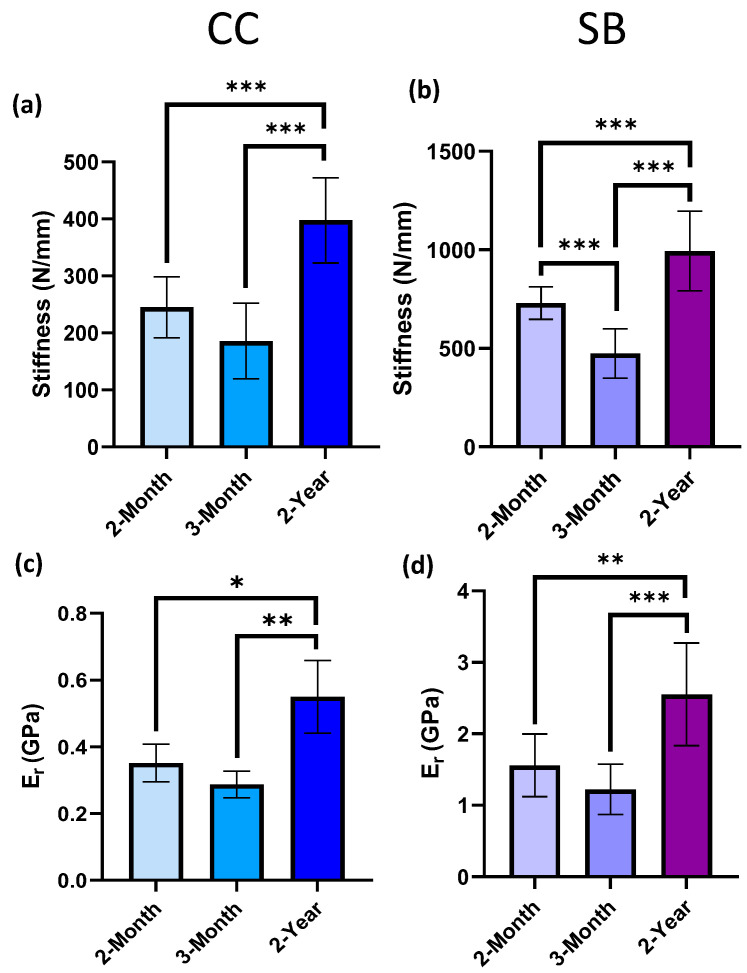
Mechanical Properties of Mineralized Regions of the Subchondral Plate: The stiffness and E_r_ of (**a**,**c**) calcified cartilage (* *p* = 0.0226; ** *p* = 0.0031; *** *p* < 0.001) and (**b**,**d**) subchondral bone (** *p* = 0.0095; *** *p* < 0.001), respectively. Data are reported as mean ± SD (*n* = 3).

The stiffness of the calcified cartilage (CC) zone significantly increased from 245.13 (±53.62) N/mm in 2-month specimens and from 185.70 (±66.46) N/mm in 3-month specimens, to 397.59 (±74.85) N/mm in 2-year specimens (Figure 4a; *** *p* < 0.001). The same trend was apparent for the E_r_ values, which significantly increased from 0.352 (±0.056) GPa at 2-months and from 0.287 (±0.040) GPa at 3-months, to 0.550 (±0.108) GPa at 2-years of age (Figure 4c; * *p* = 0.0226; ** *p* = 0.0031). In the subchondral bone, an initial 35.0% reduction in stiffness from 729.26 (±82.81) N/mm at 2-months to 473.81 (±125.19) N/mm in 3-month specimens was observed (*** *p* < 0.001; Figure 4b). Additionally, 2-year severe OA specimens had a significantly increased SB stiffness of 993.25 (±201.83) N/mm of 36.2% and 109.6% compared to both 2-month and 3-month-old specimens, respectively (*** *p* < 0.001; Figure 4b). These results were consistent with the average E_r_ values of the SB, which were also significantly higher in 2-year specimens (2.55 ± 0.71 GPa) compared to 2- month (1.56 ± 0.44 GPa, ** *p* = 0.0095) and 3-month (1.22 ± 0.35 GPa, *** *p* < 0.001) specimens (Figure 4d).

## 4. Discussion

This study demonstrates a suitable method for the characterization of mechanical properties throughout the depth of AC and in SB at the early and late stages of OA in relation to immunohistochemical markers and highlights the interrelationship between the biomechanics of AC and SB during OA initiation and progression. This study indicates that early stiffening of the deep zone of AC and early reduction in stiffness of SB may precede cartilage degeneration during OA progression.

The degradative changes observed in 2-month, 3-month, and 2 -year-old DH are consistent with other studies using similar age categories [68,82], for example, it is well documented that mild histological changes are first observed in DH guinea pigs at 3 months of age [68,72,83], with severe OA becoming apparent after 12–18 months [84]. The levels of OA correlate with increased body weight with aging, since obesity and mechanical loading are the predisposing factors that result in OA in this strain [71,85]. The validation of this animal model has already been established [86] and the pathophysiological evidence of OA is representative of those observed in human knee OA, with similarities in unilateral focal degeneration of the articular cartilage [74] and GAG loss, as well as early bone changes, including subchondral bone sclerosis, increased subchondral plate thickness, decreased porosity and increased bone mineral density [67,76,87], all of which are more pronounced on the medial side similar to human OA. Since DH guinea pigs reach skeletal maturity between 7 and 23 months of age, these would also be interesting time points to include; however, the focus of this study was to assess the very early-onset mechanical changes involved in the initiation of OA.

The nanoindentation results of this study are comparable with the ranges of the stiffness and modulus values of AC in the literature, which gradually increase throughout the AC depth [88]. In general, the elastic modulus of hyaline cartilage ranges from 1.9 to 15 MPa [89,90] and the maximum value obtained in this study for the non-calcified regions of AC (9.1 ± 0.96 MPa) is within that range. In addition, micro-indentation tests in PBS reported the average elastic modulus of the medial tibia plateau cartilage as being 2.6 ± 1.4 MPa, which corresponds to similar modulus values in the SZ and MZ in this study [91]. However, it is important to note that due to the depth-wise anisotropy and inhomogeneous structure and composition of the AC in superficial, middle, and deep zones, variation in the mechanical properties throughout the AC depth is common and can result in discrepancies in the modulus when average measurements are taken, either in bulk from indentions performed perpendicular to the cartilage surface, or when performed parallel to the surface but averaged from across all these cartilage zones [49,92]. This variation is enhanced during OA degradation where site-specific properties can be affected particularly between the early and late stages of OA. Other studies that use depth-wise measurements throughout the zonal architecture of AC, report conflicting values. For example, [93] reports E_r_ values of ~1 MPa in the SZ to ~10 MPa in the DZ from indentation tests, whereas [92] determines modulus values of 0.079 MPa in the SZ to 2.10 MPa in the DZ from compression tests. In contrast, [94] reported a much lower elastic modulus ranging from 0.020 ± 0.003 MPa in the superficial zone and up to 6.44 ± 1.02 MPa in the calcified zone of AC following nanoindentation of human specimens. Therefore, despite using a similar approach of indenting throughout the cartilage depth, disparities in the measured modulus still exist between studies depending on the scale and varying methodology.

The method used in this study allowed the detection of differences in mechanical properties throughout the depth of articular cartilage with increasing OA severity. The decrease in stiffness in the superficial layers of AC in severe OA specimens corresponds to both a decrease in proteoglycan content (shown using toluidine blue staining) and an increase in ADAMTS4 and ADAMTS5, the main proteinases responsible for aggrecan degradation that are expressed mainly in the superficial zone of AC [95,96]. This is corroborated by an AFM-based study by [97], which showed that aggrecan depletion in a mouse femur resulted in a significant decrease in the elastic modulus of cartilage from 2.0 to 0.4 MPa. However, when indenting near a free surface, particularly in the SZ, edge effects can induce artifacts in the measurements [98]. This can either be taken into account by adjusting the calculations accordingly [99], embedding the specimens in resin, or measuring the mechanical properties of AC from the cartilage surface only. However, penetration of resin into the specimen is likely to increase the sample stiffness, particularly in soft or porous tissues, whereas indention in the axial direction can alter the mechanical properties by up to 14% [43] and gives limited information only on the bulk mechanical properties of AC. In our study, not only did we detect significant differences between the stiffness of the cartilage in these zones, but we also detected changes in stiffness of the cartilage in these zones as OA progressed.

Most interestingly, the deep zone of AC underwent a significant stiffening during early OA with subsequent decrease with severe OA. This could be due to the stiffening of collagen fibrils, which occurs at the cartilage–bone interface in mildly-degraded OA cartilage [51]. This is likely since nanoscale indentation with sharp probes, such as Berkovich tips, can measure the mechanics of individual extracellular matrix components, in which collagen fibrils are an order of magnitude stiffer than proteoglycans [49,100,101]. In severe OA, this effect could have been counteracted by the aggrecan depletion observed by the increased immunohistochemical staining of ADAMTS-5 throughout the deep zone of AC and the zone of CC, during pathological tidemark advancement. In addition, matrix metalloproteinase 13 (MMP-13) expression, which is higher in late-stage compared to both early-OA or healthy cartilage, is also localized to the deep zone of AC and degrades type II collagen fibers in this zone [102,103,104]. Chondrocyte apoptosis also predominately occurs in the middle and deep zones from 7 months of age in DH guinea pigs [105] and is correlated with matrix degradation and disruption of fibrillar architecture in this region during severe OA [106]. In our study, the collagen content of the AC remained unchanged throughout OA progression, but expression was continually higher in the DZ and zone of CC, where collagen content is expected to increase in depth toward the cartilage–bone interface [107]. In the deep zone, collagen fibrils are arranged perpendicular to the joint surface and have the largest diameter in order to provide the greatest resistance to compressive forces, however, disruption in the type II collagen network is an early disease event in DH guinea pigs, which precedes histological proteoglycan loss as early as at 2 months of age [108]. Therefore, nanoindentation to detect alterations to the mechanical properties of AC and SB may be a more sensitive indicator of structural changes during early OA compared to classic histology, and including an earlier age group of DH guinea pigs (<2 months of age) would be useful for future studies for comparison. The large variation in standard deviation when the reduced modulus was averaged particularly for the deep zone is to be expected due to the sharp increase in the stiffness gradient between a depth of 120 to 140 µm in this region. This highlights the importance of step-wise analysis relating to depth from the cartilage surface as a more accurate method, rather than averaging bulk material properties of AC.

**Figure A1:**
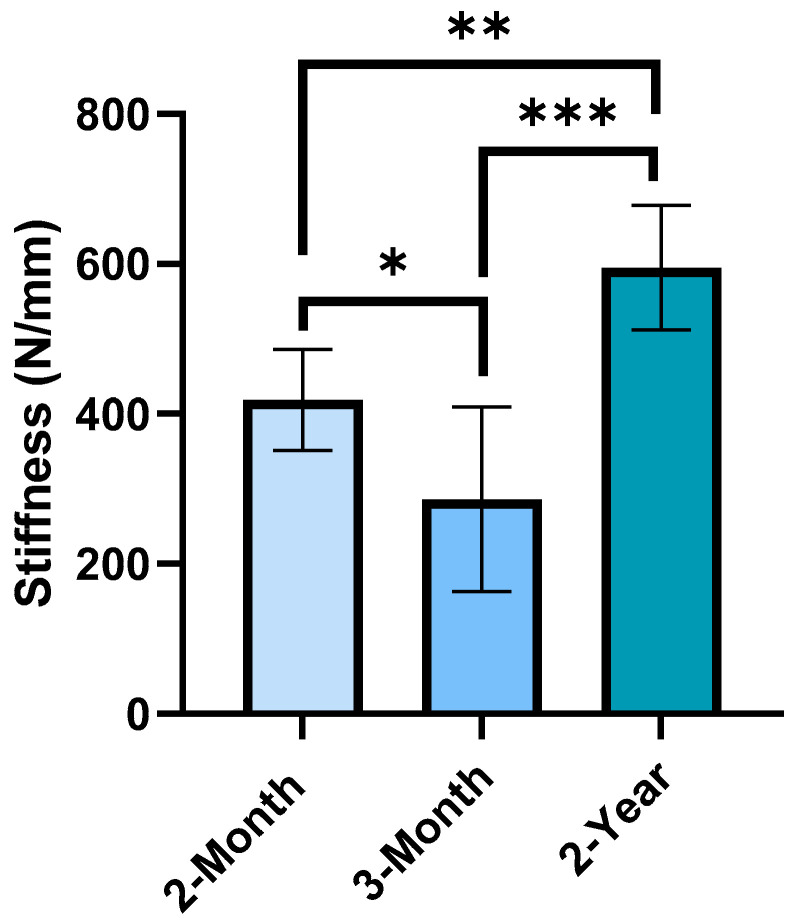
The stiffness of the subchondral plate when averaged for the calcified cartilage and subchondral bone regions (* *p* = 0.014; ** *p* = 0.0018; *** *p* < 0.001). Data are reported as mean ± SD (*n* = 3).

It is well known that during OA progression, the calcified cartilage advances into the other zones of AC causing the duplication of tidemarks and increased mineralization [5]. However, during early OA, the increased mineralization of the CC has shown to have little effect on the mechanical properties, and often no increase in modulus is detected by nanoindentation [29,39,48]. This is corroborated by the results of this study, which showed no significant differences in either the stiffness or E_r_ between 2-month and 3-month specimens. Instead, the alterations to CC relate to late-stage OA in which the effects of increased mineralization become more pronounced [109]. Despite levels of OA degradation, the modulus values for CC are comparable to those in the literature, ranging from ~0.2–0.3 GPa [30,43] but these can be as high as 15 to 25 GPa from AFM-based nanoindentation studies when embedded in resin [45,46,47]. The increase in stiffness of both CC and SB between 2-month and late-stage OA DH specimens is consistent with other similar studies in which the stiffness of the subchondral plate increased from approximately 200 N/mm, at 2-months to ~600 N/mm at 12 months but did not include an early-OA age group under 6-months of age [71]. These values are comparable to our values of 418 N/mm at 2 months, 286 N/mm at 3 months, and 594 N/mm at 2 years when averages were taken for a combination of both of these tissues (Figure A1).

The decrease in stiffness of the subchondral bone during the initial stages of OA, followed by a late-stage increase in SB stiffness and E_r_ could be attributed to early-stage remodeling, in which newly formed bone is less stiff [110], followed by a late-stage densification of SB and sclerosis [5,82]. Other nanoindentation studies observe similar trends in which there is an initial decrease in elastic modulus during early-stage OA compared to control specimens [40] and an increase in SB modulus with increasing OA grade [50]. In late-stage OA, an increase in bone mineral content, and density from 2–8 months [72], and greater subchondral bone thickness from 5 months in DH guinea pigs [67] is observed. These factors increase the stiffness and hardness of SB [41] despite the proliferation of defective bone of altered composition [111]. These microstructural changes that result in increased stiffness of SB may damage the overlying AC by increasing the strain and therefore resulting in OA progression [112] supporting the role of bone in OA initiation and progression. However, it should be noted that in our study, at the onset of OA, changes in stiffness were more apparent in cartilage than in either subchondral bone or calcified cartilage, which suggests that the cartilage may be affected first.

However, it should be noted that numerous factors can affect the mechanical properties in nanoindentation tests including tip geometry, tip size, test direction (axial or transverse testing), hydration state, and specimen preparation [43,54,64,88,113]. Therefore, making comparisons between studies utilizing different nanoindentation methods is often difficult and the reported quantitative values should be interpreted with caution. For example, the use of PMMA may be suitable for embedding hard or mineralized tissues such as SB or CC; however, infiltration of resin into soft, porous, or viscoelastic tissues such as AC may increase overall tissue stiffness [45,47], which in combination with polishing to minimize surface roughness may not be reflective of the mechanical properties of native tissue. Therefore, the use of liquid nanoindentation on unmodified tissues has its advantages, especially given that the natural surface roughness of soft materials, such as AC is unlikely to contribute to differences in contact area and load-displacement data, due to fluid flow and tissue deformation before substantial load is applied [114].

There were also limitations to this study. Firstly, a Berkovich tip was used, which is generally suitable for nanoindentation of mineralized tissues such as calcified cartilage and subchondral bone but less commonly used for soft tissues [41,47,115]. Even though Berkovich tips have been previously used in other studies to evaluate the mechanical properties of articular cartilage [34,45], blunt tips such as large-diameter spherical or flat punch tips may be better for minimizing stress concentrations and reducing contact pressures that could result in plastic deformation [31,32,116]. Secondly, the sample size in this study was also relatively small, and due to restrictions on tissue volume throughout the AC depth, a restricted number of indents were performed. Increasing both the sample size and the number of indentations [91] performed would improve the reproducibility of the study. In addition, the specimens underwent one cycle of freeze-thawing, which, in other studies, has been shown not to have a significant effect on the mechanical properties of articular cartilage [78,117,118,119,120], using fresh specimens may be more suitable for more closely approximating the precise mechanical properties of native osteochondral tissue for future nanoindentation tests. Finally, although the nanoindentation tests were performed in fluid, the test conditions were maintained at room temperature as opposed to body temperature, which may influence the force-displacement measurements and therefore may not be fully representative of a true in vivo environment [121,122,123].

In conclusion, this study uses a novel approach for liquid nanoindentation of specimens maintained in physiological saline, on unfixed tissues without the use of fixation, dehydration, or embedding for measurements of the mechanical properties of AC and SB that more closely resemble a realistic in vivo environment, that indicates that early stiffening of the deep zone of AC and early reduction in stiffness of SB may precede cartilage degeneration during OA progression.

## Data Availability

Data available on request.
